# Potentiometric sensor for iron (III) quantitative determination: experimental and computational approaches

**DOI:** 10.1186/s13065-019-0648-x

**Published:** 2019-11-18

**Authors:** Somayeh Badakhshan, Saeid Ahmadzadeh, Anoushiravan Mohseni-Bandpei, Majid Aghasi, Amir Basiri

**Affiliations:** 1 Student research committee, Kerman University of Medical Sciences, Kerman, Iran; 2 Department of Environmental Health Engineering, School of Public Health, Bam University of Medical Sciences, Bam, Iran; 3 Pharmaceutics Research Center, Institute of Neuropharmacology, Kerman University of Medical Sciences, P.O. Box: 76175-493, 76169-11319 Kerman, Iran; 4 Food, Drug and Cosmetics Safety Research Center, Kerman University of Medical Sciences, Kerman, Iran; 5 Department of Environmental Health Engineering, School of Public Health, Shahid Beheshti University of Medical Sciences, Tehran, Iran; 6 Department of Environmental Health Engineering, School of Public Health, Kerman University of Medical Sciences, Kerman, Iran; 7 Department of Chemistry, Faculty of Science, Ferdowsi University of Mashhad, Mashhad, Iran

**Keywords:** Iron (III) sensor, Environmental analyses, Benzo-18-crown-6, PVC membrane, Potentiometry

## Abstract

The current work deals with fabrication and validation of a new highly Fe^3+^ selective sensor based on benzo-18-crown-6 (b-18C6) using the potentiometric method. The proposed sensor revealed satisfactory performance for quantitative evaluation of Fe^3+^ trace amount in environmental samples. The ratio of membrane ingredients optimized and the membrane with the composition of 4:30:65.5:0.5 mg of b-18C6:PVC:o-NPOE:KTpClPB exhibited the desirable Nernstian slope of 19.51 ± 0.10 (mV per decade of activity) over the pH range from 2.5 to 5.7 with an acceptable dynamic concentration range of 1.0 × 10^−6^ M to 1.0 × 10^−1^ M and lower detection limit of 8.0 × 10^−7^ M. The proposed sensor demonstrated an appropriate reproducibility with a rapid response time of 12 s and the suitable lifetime of 10 weeks. To validate the accurate response of the proposed sensor, AAS technique applied for the determination of Fe^3+^ in real aqueous mediums such as drinking tap water and hospital wastewater sample after treatment by electrocoagulation process. Theoretical studies carried out using DFT/B3LYP computational level with 6-311G basis set to optimize the adsorption sites of Fe^+3^ cationic species by b-18C6. The obtained adsorption energy with large negative value confirmed the formation of a stable complex.
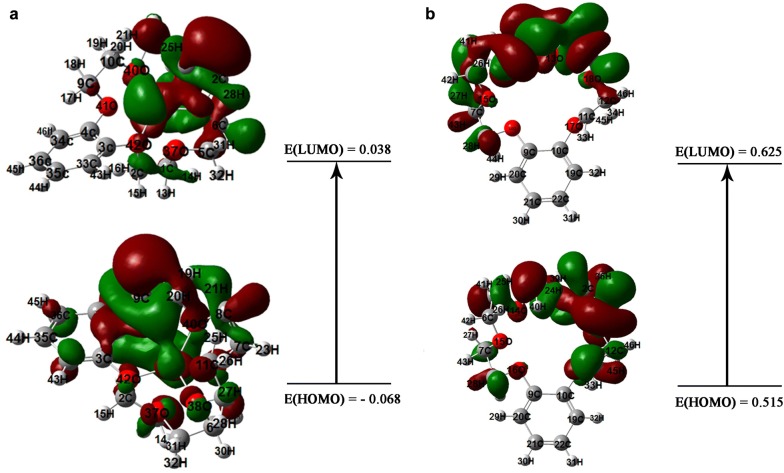

## Introduction

Iron as a heavy metal and its compounds extensively distributed in nature at various concentrations and play an important role in biological systems, especially electron transfer cycle [1–3]. Iron could pass into aqueous mediums via various procedures such as discharging of wastes, chemical corrosion of pipe which applied in the water distribution system as well as produced coagulants through electrocoagulation process using iron electrodes for the treatment of water. Iron deficiency caused anemia, while its high levels create hemochromatosis disease [2]. Iron speed up the growing of iron bacteria which get their energy from the oxidation of ferrous iron to ferric form that caused the deposition of a coating of viscous on the surface of the pipe [4]. Therefore, evaluation of iron content in the aqueous medium and developing the diagnostic tools for quantitative determination of its trace amount received extraordinary attention.

Various methods for determination of iron developed including catalytic spectrophotometric flow injection analysis [5], colorimetry [6], spectrophotometry [7], liquid–liquid microextraction [8], and ion selective electrodes [1–4, 9]. Using ion-selective electrodes (ISEs) provides a valuable tool for the determination of the specific ionic target concentration with the simultaneous existence of interfering ionic species in the aqueous medium [10]. It is noteworthy to mention that compared to instrumental techniques the new developed ISEs offer much cheaper analysis with satisfactory dynamic concentration range and detection limit [11, 12]. Due to the considerable advantages of ISEs including simple preparation and application, superior reproducibility with high selectivity and high-speed response, they received high attention for environmental, agricultural and industrial analysis [13–15].

**Fig. 1:**
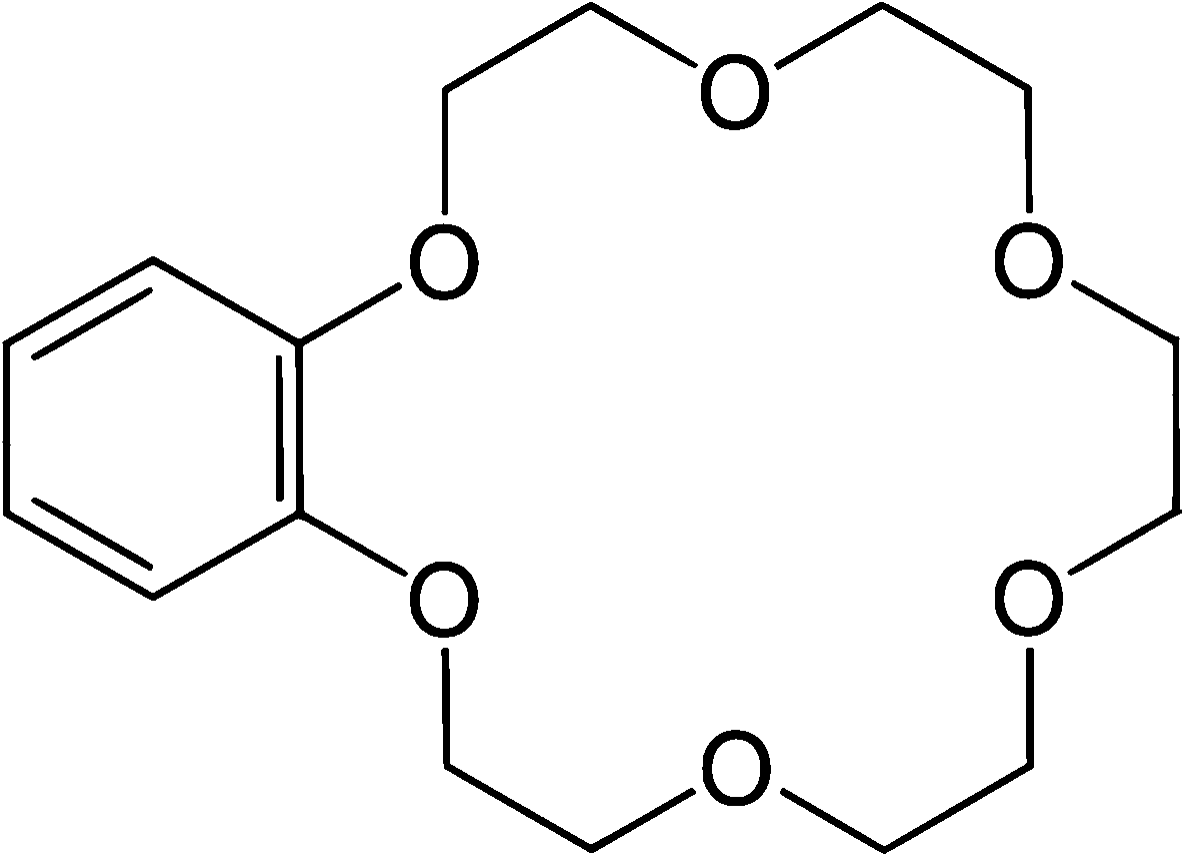
Chemical structure of benzo-18-crown-6

Crown ethers; received extraordinary attention by many research groups as an important category of macrocyclic host molecules for the last two decades. Benzo-18-crown-6 is a versatile receptor with a three-dimensional structure which makes it capable to form a stable complex with high selectivity and efficient binding properties towards Fe^3+^ as the target ion [16, 17]. Host–guest chemistry of benzo-18-crown-6 which makes it a suitable ionophore for constructing the current potentiometric sensor includes the weak and reversible nature of non-covalent intermolecular interactions with Fe^3+^ ions through coordination bonds between the donor atoms of oxygen with the Fe^3+^ ions, as well as π-coordination of the Fe^3+^ ions with the benzene ring [18] (see Fig. 1).

It is noteworthy to mention that, the polymeric membrane is the most considerable issue in fabrication of ion selective electrode. It consists of polymeric matrix, ionophore, plasticizer and lipophilic additive. The polymeric membrane physically separates the internal filling solution from the sample solution and it is the source of the signal generated by the ISE. The nature and amount of each component have a significant effect on the characteristics performance of the ISE.

Many polymers have been utilized as a matrix in fabrication of ion selective membranes which provide considerably good mechanical strength properties and structural integrity for the membrane [19]. High molecular weight polyvinyl chloride (PVC) with high glass transition temperature (Tg = 80 °C) is a brittle polymer at ambient conditions, hence using of plasticizer is unavoidable to reduce the value of the Tg. The ionophore is a macrocyclic compound which can selectively bind to the target ion and act as ion carrier for transferring ions from aqueous phase into the polymeric membrane phase. The complex formation constant between ionophore and target ion must be high enough to provide a complex with considerable selectivity in membrane phase. However, the value of the mentioned constant should not be so large in order to avoid the formation of kinetically irreversible complex [20].

Plasticizer plays an important role on optimizing the physical properties of the membrane by reducing the high glass transition temperature of PVC, as well as increasing the mobility of the active species and enhancing the flexibility of the polymer chain [21]. Also, it provides a good ionic conductivity under the ambient conditions which allow the diffusion of the membrane components into a homogenous lipophilic environment. Moreover, the nature of plasticizer significantly affected the selectivity and measuring range of ISEs [22]. It is known that the addition of proper lipophilic additive to membrane demonstrates a significant improve in the characteristic performance of ion selective electrode such as selectivity, stability and response time. Besides, it reduce the membrane impedance [20]. Even though the presence of ion exchangers in the membrane provides beneficial effects, but the excess amount of it declines the electrode performance [23].

The literature review revealed that the recently developed iron (III) selective electrodes suffered from undesirable characteristics such as low selectivity with narrow solution pH range as well as limited concentration range and lengthy runtime [1–4, 9]. Therefore, herein, a new iron (III) selective electrode with satisfactory characteristic performance developed and successfully applied for evaluation of iron (III) content in various aqueous mediums.

## Experimental

### Reagents

All chemicals are analytical grade and used without any additional refinement. Polyvinyl chloride (PVC) with high molecular weight, benzo-18-crown-6 (b-18C6) and all investigated salts (nitrate and chloride) purchased from Merck company. Sodium tetraphenylborate (NaTPB), 2-nitrophenyl octyl ether (o-NPOE), tetradodecylammonium tetrakis(4-chlorophenyl)-borate (TDATpClPB), nitrobenzene (NB), Dioctyl phthalate (DOP), potassium tetrakis(4-chlorophenyl)borate (KTpClPB), dibutyl phthalate (DBP), tetrahydrofuran (THF), and sodium tetrakis [3,5-bis (trifluoromethyl) phenyl] borate (NaTFPB) obtained from Sigma-Aldrich, and Scharlau company. For pH adjustments, hydrochloric acid (HCl) and sodium hydroxide (NaOH) used. Hydrogen peroxide (H_2_O_2_) which used as an oxidant to oxidize Fe(II) to Fe(III) in real samples purchased from Merck. De-ionized water used to prepare all daily solutions.

### Apparatus and potential measurement

Metrohm 827 pH/mv meter applied for the potential measurements using Metrohm Ag, AgCl/3 M KCl reference electrodes and Mettler Toledo pH electrode at 25 °C. A Varian Atomic Absorption Spectrometry (Model AA240) and Optizen UV–Visible spectrophotometer (model 3220UV) employed for validation of sensor response and evaluation of complex reaction between the ionophore and iron (III), respectively. SEM (Philips model XL30E SEM) and FT-IR (Bruker model Alpha) were used to validate, identify and characterization of the synthesized membrane. A de-ionized water system (Smart2Pure model TKA) to supply water requirements was used throughout the study. The following electrochemical call employed for EMF measurements:

Ag/AgCl ref. electrode, KCl (3 M)║sample solution│selective polymeric membrane│ 1.0 × 10^−3^ M Fe(NO_3_)_3_ standard solution║ Ag/AgCl ref. electrode, KCl (3M).

### Electrode and real sample preparation

The amount of 4.0 mg b-18C6, 30.0 mg PVC, 65.5 mg o-NPOE, and 0.5 mg KTpClPB dissolved in 3 ml THF to prepare the polymeric membrane. The prepared solution concentrated until a viscose mixture achieved and afterward employed for preparing a polymeric membrane with the thickness of 0.3 mm at the end of the Pyrex tube with 3 mm i.d that finally filled with the 1.0 × 10^−3^ M Fe(NO_3_)_3_ standard solution.

To analyze the drinking tap water and hospital wastewater which treated through the electrocoagulation process, the standard addition method applied. For oxidizing iron (II) into iron (III) a mix of 5 mL H_2_O_2_ and 5 mL of HNO_3_ both 1 N added to the real samples.

### Computational methods

Theoretical studies carried out using DFT/B3LYP computational level with 6-311G basis set to optimize the adsorption sites of Fe^+3^ cationic species by b-18C6 using the Gaussian 09 program package. Natural bond orbital (NBO) analysis carried out to investigate the strength and nature of the intermolecular interactions of the formed complex. The charge distribution on the structure of the formed complex investigated.

## Results and discussion

### Potentiometric Response and membrane optimization process

**Fig. 2:**
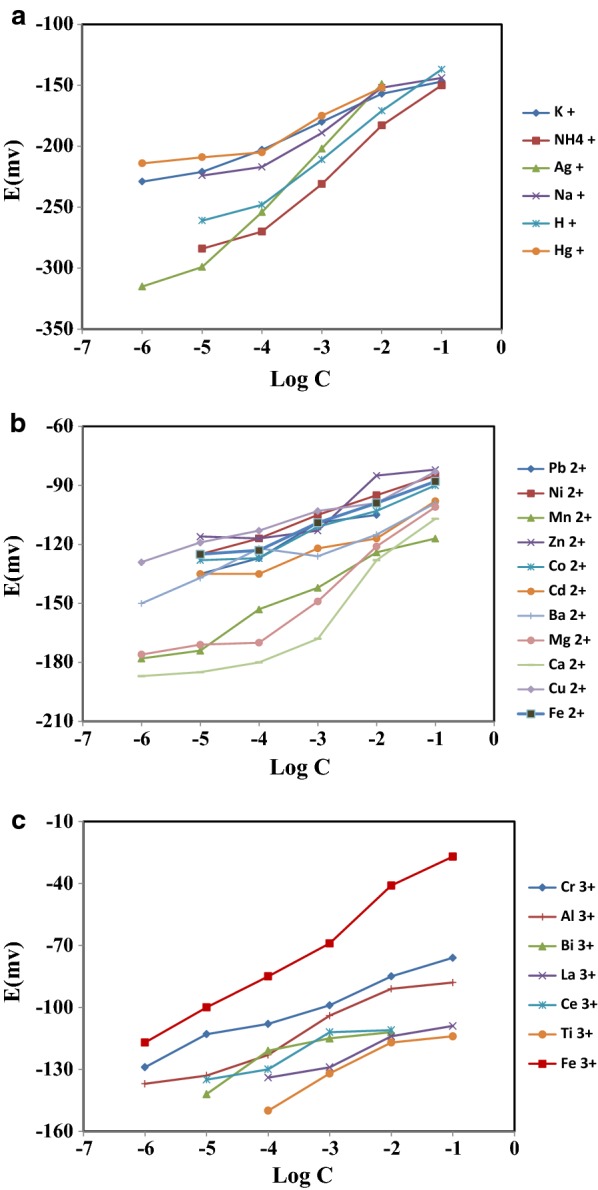
The potential responses of PVC membrane sensor based on b-18C6 towards monovalent cations (**a**), bivalent cations (**b**), and trivalent cations (**c**) at 25 °C

The responses of the proposed sensor towards various ions investigated over the wide concentration variety and showed in Fig. 2. The obtained results indicated a preferred complex formation between b-18C6 and Fe(III) in comparison to the other investigated ions with the satisfactory Nernstian slope of 19.51 mV per decade of activity. The observed behaviour may be attributed to the rapid exchange kinetics of the generated complex [24, 25].

It can be concluded from the characteristic performance of the proposed electrode, membrane ingredients significantly affected the Nernstian response. As seen from the Table 1, besides the importance of ionophore nature in constructing a selective membrane sensor, the sensitivity and selectivity of the ISEs are significantly affected by the composition of membrane [26]. Therefore, effects of various parameters such as different amount of membrane ingredients as well as different type and amount of plasticizers and lipophilic additives on the potential responses of fabricated selective electrodes were investigated.

**Table 1:** Optimization of the membrane composition to fabricate high iron (III) selective electrode

Sensor no.	I	PVC	Plasticizer				Lipophilic additive				Nernstian slope (mV decade^−1^)^a^	R^2^	Response time (s)
			DOP	DBP	o-NPOE	NB	KTpClPB	NaTFPB	TDATpClPB	NaTPB			
1	0	30.0		–	69.5	–	0.5	–	–	–	4.68 ± 0.21	0.866	N/A
2	3.0	30.0	–	–	66.5	–	0.5	–	–	–	16.51 ± 0.25	0.998	17
3	4.0	30.0	–	–	65.5	–	0.5	–	–	–	19.51 ± 0.10	0.998	12
4	5.0	30.0	–	–	64.5	–	0.5	–	–	–	24.23 ± 0.36	0.996	10
5	4.0	30.0	–	–	65.5	–	–	0.5	–	–	18.14 ± 0.41	0.940	13
6	4.0	30.0	–	–	65.5	–	–	–	0.5	–	12.30 ± 0.48	0.933	17
7	4.0	30.0	–	–	65.5	–	–	–	–	0.5	17.77 ± 0.35	0.970	15
8	4.0	30.0	65.5	–	–	–	0.5	–	–	–	14.40 ± 0.54	0.987	15
9	4.0	30.0	–	65.5	–	–	0.5	–	–	–	17.40 ± 0.45	0.992	14
10	4.0	30.0	–	–	–	65.5	0.5	–	–	–	11.62 ± 0.39	0.973	17
11	4.0	30.0	–	–	65.8	–	0.2	–	–	–	18.34 ± 0.26	0.998	15
12	4.0	30.0	–	–	65.0	–	1	–	–	–	22.17 ± 0.33	0.975	15
13	4.0	30.0	–	–	64.5	–	1.5	–	–	–	24.37 ± 0.43	0.975	13

^a^Average and standard deviation for triplet measurements

The literature surveys were in accordance with the acquired results, which indicated that the characteristic performance of the developed sensor in the term of sensitivity and selectivity affected meaningfully by changing the amount of employed ionophore [27]. In addition, it found that the membrane containing o-NPOE as a plasticizer with higher dielectric constant exhibited the best Nernstian response which is perhaps due to the facilitating the Fe^+3^ extraction from aqueous solution to the membrane phase [28, 29]. Among various anion excluder used in the current work, KTpClPB demonstrated better linear range with an acceptable Nernstian slope. Lipophilic additives diminished the anionic interference effects and by decreasing the ohmic resistance of the membrane enhanced the cation extraction process [29, 30].

In order to improve the performance of the proposed sensor, standard internal solutions with different concentration introduced to the working electrode. The standard internal solution of 1.0 × 10^−3^ M Fe(NO_3_)_3_ demonstrated the best electrochemical performance which used for further studies. The mentioned concentration used for equilibrating the electrodes over the night (Table 2).

### Sensor characterization

**Fig. 3:**
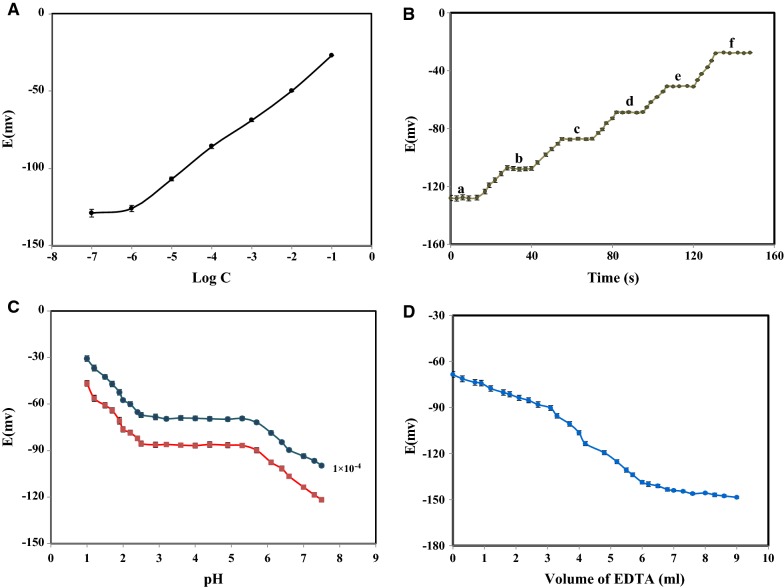
The Fe^3+^ selective electrode characterization; **A** Calibration graph, **B** The dynamic response time of the for step changes in the concentration of iron solution: (a) 1.0 × 10^−6^ M, (b) 1.0 × 10^−5^ M, (c) 1.0 × 10^−4^ M, (d) 1.0 × 10^−3^ M, (e) 1.0 × 10^−2^ M and (f) 1.0 × 10^−1^ M. **C** The pH effect of the sample solutions on the potential response. **D** Potentiometric titration curve of iron ion (1.0 × 10^−3^ M, 50 mL) with standard EDTA solution (1.0 × 10^−2^ M) using fabricated electrode based on b-18C6 as an indicator electrode at 25 °C

To generate the calibration curve, the proposed electrode was conditioned in 1.0 × 10^−2^ M solution of the Fe^3+^ ions for 24 h. The potential responses of fabricated sensor over a very wide concentration range of 1.0 × 10^−8^ M to 1.0 × 10^−1^ M were obtained and showed in Fig. 3A. The proposed electrode demonstrated an acceptable performance over the examined centration range. The value of 19.51 ± 0.10 mV per decade of activity and 8.0 × 10^−7^ M found as its repeatable slope and detection limit, respectively.

Repeatability of the developed sensor investigated and the obtained slope was found to be 19.51 ± 0.10 mV per decade of activity. Moreover, the reproducibility parameter for the proposed sensor revealed a satisfactory Nernstian slope of 19. 44 ± 0.28 mV per decade of activity. In accordance with the SEM studies, the variation in the morphology and thickness of the fabricated polymeric membranes may result in small changes in the extraction equilibrium of target ion at the interface of sample solution aqueous layer and fabricated membrane [31].

**Table 2:** Characterization of proposed iron (III) sensor in the term of repeatability and reproducibility

Study	Nernstian slope (mV decade^−1^)	Average	Standard deviation	*RSD*
Repeatability	19.46, 19.43, 19.39 19.53, 19.69, 19.47 19.58	19.51	0.10	0.52
Reproducibility	18.95, 19.25, 19.31 19.63, 19.74, 19.47 19.72	19.44	0.28	1.48

The dynamic response time as a significant operating parameter for the performance evaluation of the developed sensor investigated and it found to be about 12 s. The observed behaviour attributed to the fast exchange kinetics of complexation-decomplexation process between investigated cation at the boundary of the polymeric selective membrane with the examined solution (see Fig. 3B).

To investigate the stability of the membrane and lifetime of the proposed sensor, they were used daily for at least 2 h and the obtained slopes summarized in Additional file 1. The obtained slopes indicated that the proposed sensor could be used practically for almost 10 weeks. It found that the observed slope changed from the initial value of 19.51 ± 0.10 to the final value of 18.57 ± 0.44 mV per decade of activity after 10 weeks which probably attributed to the leaching of membrane ingredients [32].

The potential response of the proposed sensor was found to be pH independent over the satisfactory range of 2.5 to 5.7 (see Fig. 3C). The drift at pH lower than 2.5 probably attributed to the high concentration of H_3_O^+^ ions which cooperate in process of charge transport of the fabricated membrane. Whereas at pH value higher than 5.7, because of hydroxyl complexes formation the observed potential response of the developed sensor changed [33, 34].

On the other hand, the proposed sensor employed for potentiometric titration of Fe(NO_3_)_3_ solution by EDTA standard solution. The obtained titration curve revealed a successful titration process with a standard sigmoidal shape indicates the stoichiometry of 1:1 for the formed EDTA- Fe^3+^ complex (see Fig. 3D).

Separate solution method (SSM) applied to evaluate the selectivity coefficient of the proposed sensor. The applied concentration for both of analyte and interfering ions adjusted at 1.0 × 10^−2^ M. As seen from Table 3, the selectivity coefficient for examined interfering ions was found to be in the order of 10^−3^ to 10^−5^. The obtained values showed that in comparison to the earlier described iron (III) sensor a considerable improvement observed [2, 3].

**Table 3:** Proposed iron (III) sensor selectivity coefficient on separate solution method

Examined Cation	Log $K_{Cs,M}^{Pot}$	$K_{Cs,M}^{Pot}$
Fe^3+^	1	0
K^+^	− 4.07	8.47 × 10^−5^
H^+^	− 3.56	2.74 × 10^−4^
Na^+^	− 3.93	1.15 × 10^−4^
NH_4_^+^	− 4.21	6.22 × 10^−5^
Ag^+^	− 4.18	6.57 × 10^−5^
Hg^+^	− 4.36	4.36 × 10^−5^
Zn^2+^	− 2.28	5.23 × 10^−3^
Ni^2+^	− 2.43	3.75 × 10^−3^
Ba^2+^	− 3.12	7.49 × 10^−4^
Cd^2+^	− 3.06	8.65 × 10^−4^
Co^2+^	− 2.75	1.78 × 10^−3^
Cu^2+^	− 2.31	4.86 × 10^−3^
Mn^2+^	− 4.05	8.94 × 10^−5^
Pb^2+^	− 3.42	3.84 × 10^−4^
Mg^2+^	− 3.27	5.42 × 10^−4^
Fe^2+^	− 2.81	1.58 × 10^−3^
Ca^2+^	− 3.58	2.61 × 10^−4^
Cr^3+^	− 2.49	3.26 × 10^−3^
Ce^3+^	− 4.26	5.43 × 10^−5^
La^3+^	− 4.17	6.75 × 10^−5^
Ti^3+^	− 4.35	4.43 × 10^−5^
Bi^3+^	− 4.30	5.01 × 10^−5^
Al^3+^	− 3.10	7.89 × 10^−4^

### Membrane characterization

**Fig. 4:**
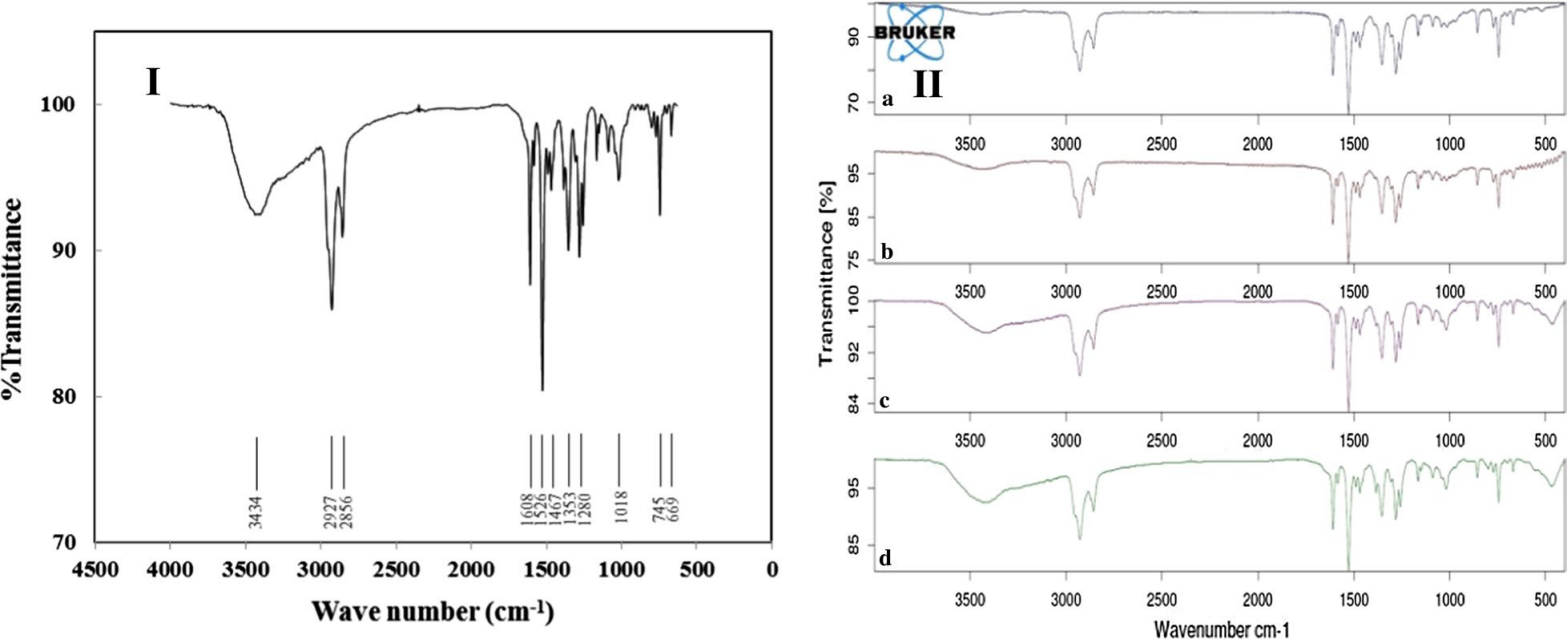
FT-IR spectra of the fabricated Fe^3+^ selective membrane (**I**) and comparison of FT-IR spectra for membrane ingredients under different conditions (**II**): a newly dry fabricated membrane, b membrane saturated in iron solution for 24 h, c used membrane after 5 weeks and d used membrane after 15 weeks

In the current work the FT-IR investigation employed as qualitative technique to evaluate the prepared membrane in the term of lifetime (see Fig. 4I). Evidently, the existence of b-18C6 in membrane composition confirmed by the strong absorption band appeared in 1280 cm^−1^ frequency due to C–O sp^3^ stretching. The presence of o-NPOE deduced by its two strong absorption bands associated with the nitro functional group (NO_2_) including an asymmetric and a symmetric stretching vibration appear at 1526 cm^−1^ and 1353 cm^−1^, respectively. Moreover, the observed absorption bands at 745 cm^−1^ and 1081 cm^−1^ which related to C–Cl and B–C stretching, respectively, confirmed the presence of lipophilic additive KTpClPB. Lastly, the presence of PVC as an inert matrix of the membrane confirmed by gauche absorption bands observed in 669 cm^−1^ region. On the other hand, two absorption bands in 1608 cm^−1^ and 1467 cm^−1^ frequencies attributed to aromatic stretching of C=O, one absorption band around 2859 cm^−1^ related to C–H sp^3^ stretching, and stretching of =C–H sp^2^ appears at 2927 cm^−1^ are some general absorption bands for all membrane ingredients. The wide absorption band related to O–H stretching observed in 3434 cm^−1^ owing to the membrane saturation in the aqueous medium. As it is illustrated in Fig. 4II, the evaluation of the spectra related to the employed membranes demonstrated that no significant change observed in the membrane composition which proved its long lifetime and stability.

**Fig. 5:**
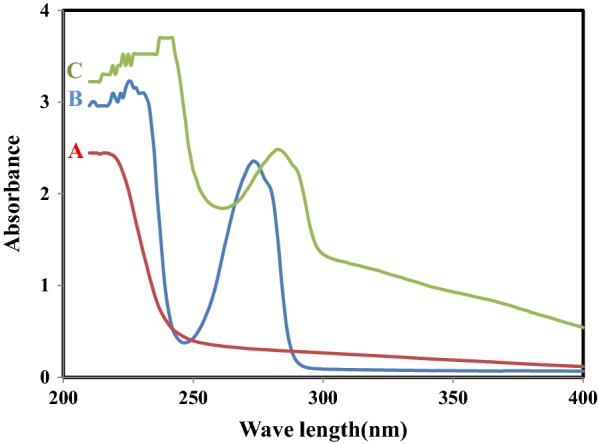
UV–Vis absorption spectra: (A) 1.0 × 10^−3^ M Fe^3+^, (B) 1.0 × 10^−3^ M b-18C6 and (C) mixture of ionophore and cation

To examine the preferred coordination of Fe^3+^ ions by b-18C6, UV–Vis spectroscopy analysis carried out. Literature surveys revealed that the ionic species with higher affinity to form complex, resulted in more significant variation in appearance and position of the obtained spectrum [35]. The UV spectroscopy analysis practiced for the b-18C6, Fe^3+^ cation and their 1:1 mixture and demonstrated in Fig. 5. As seen, the ionophore and Fe^3+^ displayed two separate maximum absorptions at 273 nm and 215 nm, respectively. Whereas for their mixture, the intensity of the observed absorption band increased slightly and shifted to 282 nm. The observed behaviour attributed to the preferred complexation between b-18C6 and Fe^3+^ cation.

To image the surface characteristics of the proposed selective membrane such as fouling and swelling, SEM investigations were carried out at 10 µm magnifications [36].

**Fig. 6:**
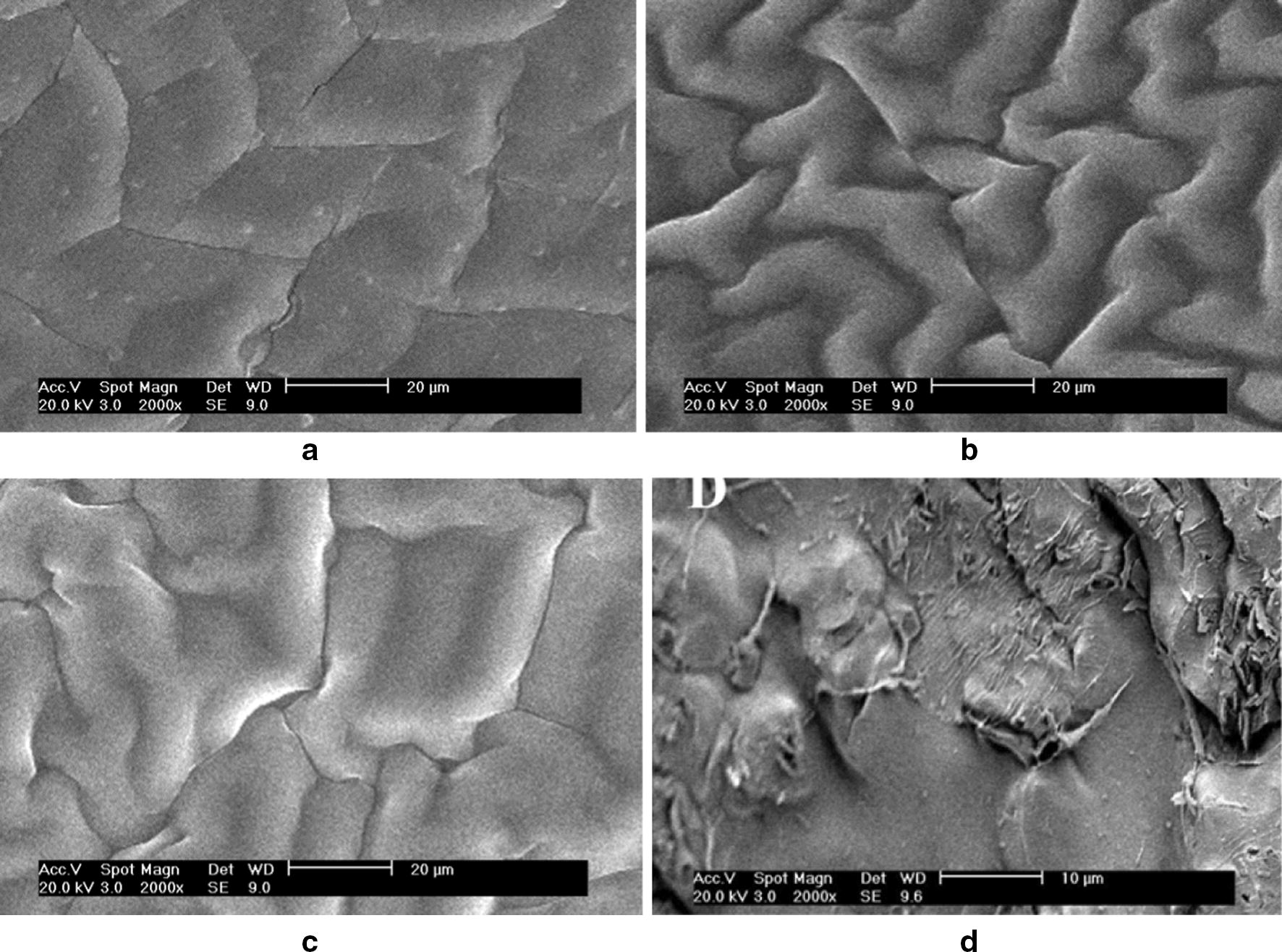
Scanning electron microphotographs (SEM) of iron (III) selective PVC membrane at 10 µm magnifications: **a** membrane without ionophore that conditioned for 1 day, **b** membrane with ionophore that conditioned for 1 day, **c** membrane that conditioned for 10 weeks, and **d** membrane that conditioned for 20 weeks and used daily over the mentioned period

As seen from Fig. 6, lack of ionophore in the prepared membrane resulted in a physically tight structure, however in the case of its adding to the membrane a physically permeable and loose structure observed. Furthermore, due to the daily usage of the proposed electrode, the membrane shows a swollen structure over 10 weeks. On the other hand, the surface of the membrane completely covered by contaminated sediments after daily usage over 20 weeks and it lost its ability.

### Sensor validation

Drinking tap water and hospital wastewater sample after treatment through the electrocoagulation process used as environmental samples to evaluate the analytical applicability and accuracy of the proposed sensor. As seen from Table 4, acceptable compliance observed between the results achieved by the proposed sensor and the data acquired from atomic absorption spectrometry (AAS).

**Table 4:** Determination of iron (III) amount of real samples using standard addition technique

Sample	Amount added (ppm)	Concentration of Fe^3+^ ions (ppm)		Recovery (%)
		ISE method^a^	AAS method^a^	
Drinking tap water	–	< Limit of detection	< Limit of detection	N/A
	5.00	5.21 ± 0.32	5.08 ± 0.11	102.5
	10.00	10.03 ± 0.19	10.13 ± 0.15	99.0
	15.00	15.32 ± 0.41	15.25 ± 0.42	100.4
	25.00	25.39 ± 0.24	25.10 ± 0.28	101.1
	40.00	39.85 ± 0.36	40.37 ± 0.17	98.7
Treated hospital waste water	–	14.11 ± 0.21	13.73 ± 0.21	102.7
	10.00	23.85 ± 0.34	23.52 ± 0.32	101.4
	20.00	33.21 ± 0.19	33.84 ± 0.22	98.1
	30.00	43.79 ± 0.35	43.44 ± 0.36	100.8

^a^Average and standard deviation for triplet measurements

### Computational analysis

The adsorption process of Fe^+3^ cationic species by b-18C6 theoretically investigated employing the DFT/B3LYP computational level with the 6-311G basis set using the Gaussian 09 program package. The following equation expressed the calculation procedure of E_ads_ (adsorption energy) between Fe^+3^ and b-18C6:

1 $${\text{E}}_{\text{ads}} = {\text{ E}}_{{({\text{complex}})}} - {\text{ E}}_{{({\text{b}} - 1 8 {\text{C6}})}} - {\text{ E}}_{{\left( {{\text{Fe}}^{ + 3} } \right)}}$$

where E _(complex)_, E _(b-18C6)_ and E _(Fe_^+3^_)_ denote the total energy of the formed complex, the total energy of b-18C6, and the total energy of Fe^+^ cationic species, respectively.

**Fig. 7:**
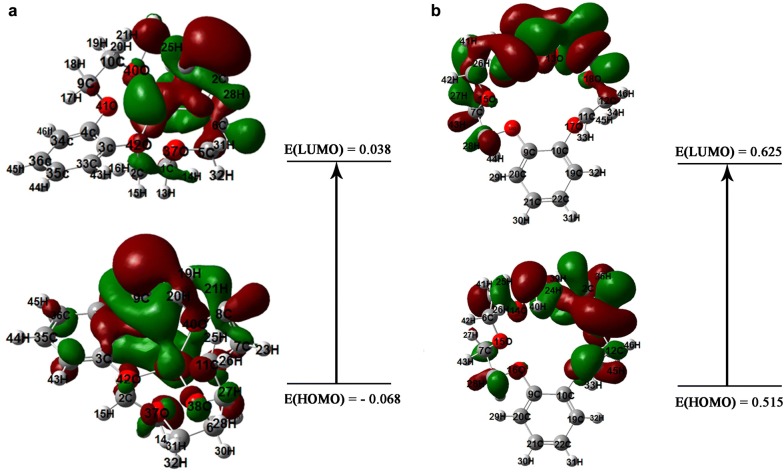
HOMO and LUMO compositions of the frontier orbital for **a** formed complex and **b** on benzo-18-crown-6 ionophore

According to the calculated adsorption energy of − 19.04 eV which is a large negative value can be concluded that the optimized structure of the formed complex is stable. By evaluating the gap of energy between the highest occupied molecular orbital (HOMO) and the lowest unoccupied molecular orbital (LUMO) the molecular electrical conductance properties can be described. The HOMO–LUMO diagrams and the energies of the formed complex and b-18C6 demonstrated in Fig. 7. The HOMO–LUMO energy gap of 0.107 eV found for the formed complex which indicated adequate electron conductivity due to the low difference between molecular orbitals. Moreover, the obtained result provided a measure of structural stability properties [37].

Natural bond orbital (NBO) analysis carried out to study the strength and nature of the intermolecular interactions of the formed complex at the same level of theory. The obtained results including some important orbital interactions of oxygen atoms which contributed in donor–acceptor interactions with LP* (Fe) as well as their second-order perturbation stabilization energies E^(2)^ summarized in Table 5. The achieved results highlighted that iron prefers to participate in the complex formation process as the acceptor while the aromatic ring and oxygen prefer to participate as donors through coordinate bands.

**Table 5:** NBO analysis for some significant orbital interactions of the formed complex

Donor atom-number	Acceptor	E (2) (kcal/mol)
O-37	LP^*^ Fe	0.36
O-38	LP^*^ Fe	0.73
O-39	LP^*^ Fe	0.81
O-40	LP^*^ Fe	0.72
O-41	LP^*^ Fe	1.68
O-42	LP^*^ Fe	0.21

**Fig. 8:**
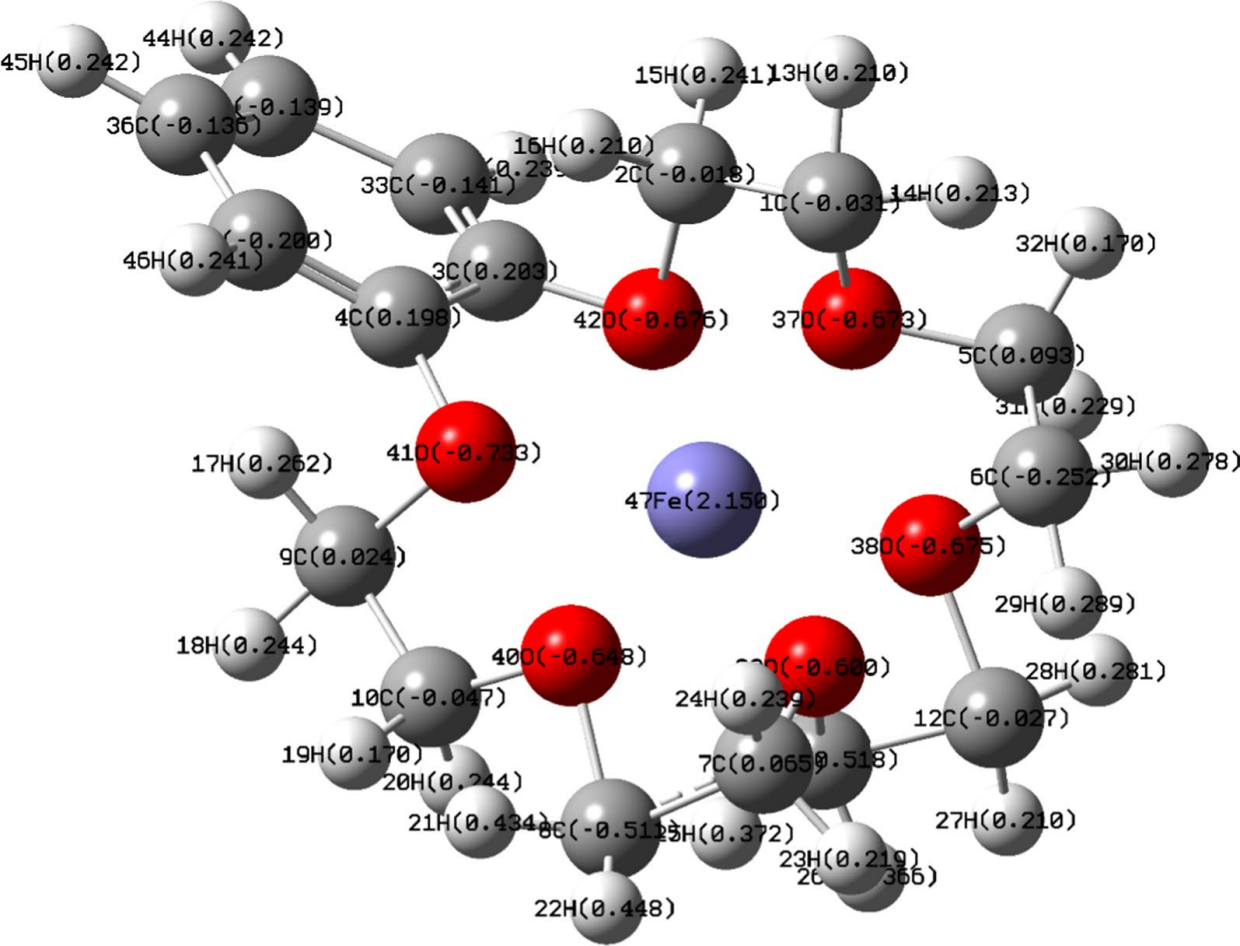
Charge distribution of the optimized structure of formed complex

The charge distribution on the structure of the formed complex demonstrated in Fig. 8. According to the calculated value in Table 5, for second-order perturbation stabilization energies, oxygen atom number 41 has the largest E^(2)^ energy and highest interaction level with iron (III) cation. The charges of selected oxygen atoms and iron atom summarized in Additional file 2.

### Comparison of reported works with proposed sensor

The characteristics performance of the proposed sensor in comparison to the early described Fe^3+^ ion-selective sensors summarized in Table 6. The developed sensor in the current work revealed better characteristics in the terms of dynamic range, the lower limit of detection and response time. Since the potential response of the fabricated sensor in the current work was independent of the solution pH over a reasonable range, it could be employed as promising tool for analysis of iron (III) concentration in environmental fields.

**Table 6:** Comparison of the characteristics performance of fabricated iron (III) selective electrode in this study with reported iron (III) electrodes

Ionophore	Working range (M)	Detection limit (M)	Slope (mV decade^−1^)	Response time (s)	pH	Refs.
Bis-bidentate Schiff (BBS)	1.0 × 10^−7^ to 1.0 × 10^−2^	7.4 × 10^−8^	19.3 ± 0.6	< 15	1.9–5.1	[2]
µ-bis(tridentate)	6.3 × 10^−6^ to 1.0 × 10^−1^	5.0 × 10^−6^	20.0	15	3.5–5.5	[1]
2-[(2-hydroxy-1-propenyl-buta-1,3-dienylimino)-methyl]-4-*p*-tolylazo-phenol [HPDTP]	3.5 × 10^−6^ to 4.0 × 10^−2^	(2.5 ± 0.5) × 10^−6^	28.5 ± 0.5	15	4.5–6.5	[4]
4-amino-6-methyl-3-methylmercapto-1,2,4-triazin-5-one (AMMTO)	1.0 × 10^−6^ to 1.0 × 10^−1^	6.8 × 10^−7^	19.4 ± 0.5	< 15	2.2–4.8	[9]
N(2hydroxyethyl)ethylenediamine-N, N′, N″-triacetic acid (NTA)	1.0 × 10^−9^ to 1.0 × 10^−2^	3.0 × 10^−10^	19.5 ± 0.4	10	1.8–4.5	[3]
Current study	1.0 × 10^−6^ to 1.0 × 10^−1^	8.0 × 10^−7^	19.51 ± 0.10	12	2.5–5.7	**–**

## Conclusions

The developed highly Fe^3+^ selective electrode in the current work revealed an acceptable performance as a diagnostic tool for the evaluation of trace amount of iron (III) in drinking tap water and treated hospital wastewater samples. The characteristic performance of the proposed sensor is favorable compared to previously developed iron (III) potentiometric sensors. The theoretical studies through density functional theory confirmed the preferred coordination between Fe^3+^ cation and b-18C6. The obtained computational results established their stable and selective interaction.

## Supplementary information

**Additional file 1.** The life time of the proposed iron (III) sensor.

**Additional file 2.** Charges of oxygen and iron atoms in the formed complex.

## Data Availability

All data and materials could be available upon the request.
