# Supplementary material for: Potentiometric sensor for iron (III) quantitative determination: experimental and computational approaches
Source: BMC Chem. 2019 Nov 18;13(1):131. doi: 10.1186/s13065-019-0648-x (PMC6859631; doi:10.1186/s13065-019-0648-x)
Supplement: Supplementary file 1 — Additional file 1. The life time of the proposed iron (III) sensor. [file 13065_2019_648_MOESM1_ESM.docx]

**Additional file 1:** The life time of the proposed iron (III) sensor.

| **Period (weeks)** | **Nernstian slope***  **(mV decade^-1^)** | **Detection**  **limit (M)** |
| --- | --- | --- |
| 1 | 19.51±0.10 | 8.0×10^-7^ |
| 2 | 19.44±0.35 | 8.0×10^-7^ |
| 3 | 19.31±0.15 | 9.0×10^-7^ |
| 4 | 19.19±0.34 | 9.5×10^-7^ |
| 5 | 19.11±0.25 | 9.5×10^-6^ |
| 6 | 18.98±0.19 | 1.0×10^-6^ |
| 7 | 18.76±0.37 | 1.0×10^-6^ |
| 8 | 18.78±0.27 | 1.0×10^-6^ |
| 9 | 18.63±0.36 | 1.0×10^-6^ |
| 10 | 18.57±0.44 | 1.5×10^-6^ |
| 11 | 18.47±0.43 | 2.5×10^-6^ |
| 12 | 18.21±0.56 | 4.0×10^-6^ |
| 13 | 17.86±0.53 | 5.5×10^-6^ |

***** Average and standard deviation for quadruplet measurements.
