# Supplementary material for: Potentiometric sensor for iron (III) quantitative determination: experimental and computational approaches
Source: BMC Chem. 2019 Nov 18;13(1):131. doi: 10.1186/s13065-019-0648-x (PMC6859631; doi:10.1186/s13065-019-0648-x)
Supplement: Supplementary file 2 — Additional file 2. Charges of oxygen and iron atoms in the formed complex. [file 13065_2019_648_MOESM2_ESM.docx]

Additional file 2: Charges of oxygen and iron atoms in the formed complex.

| **charge** | **Atom-Number** |
| --- | --- |
| -0.673 | Oxygen-37 |
| -0.675 | Oxygen-38 |
| -0.600 | Oxygen-39 |
| -0.648 | Oxygen-40 |
| -0.733 | Oxygen-41 |
| -0.675 | Oxygen-42 |
| 2.150 | Iron-47 |
